# Management of Pneumothorax in Emergency Medicine Departments: Multicenter Trial

**DOI:** 10.5812/ircmj.11586

**Published:** 2013-12-05

**Authors:** Abdulkadir Ince, Dogac Niyazi Ozucelik, Akkan Avci, Ozgur Nizam, Halil Dogan, Mehmet Ali Topal

**Affiliations:** 1 Taksim Ilk Yardim Training and Research Hospital, Adana, Turkey; 2 Bakirkoy Dr. Sadi Konuk Training and Research Hospital, Adana, Turkey; 3 Adana Numune Training and Research Hospital, Adana, Turkey; 4 Okmeydani Training and Research Hospital, Adana, Turkey

**Keywords:** Emergency Medicine, Pneumothorax, Thoracostomy

## Abstract

**Background:**

Pneumothorax is common and life-threatening clinical condition which may require emergency treatment in Emergency Medicine Departments.

**Objectives:**

We aimed to reveal the epidemiological analysis of the patients admitted to the Emergency Department with pneumothorax.

**Material and Methods:**

This case-control and multi-center study was conducted in the patients treated with the diagnosis of pneumothorax between 01.01.2010-31.12.2010. Patient data were collected from hospital automation system. According to the etiology of the pneumothorax, study groups were arranged like spontaneous pneumothorax and traumatic pneumothorax.

**Results:**

82.2% (n = 106) of patients were male and 17.8% (n = 23) of patients were female and mean age were 31.3 ± 20,2 (Minimum: 1, Maximum: 87). 68.2% (n = 88) of patients were spontaneous pneumothorax (61.36%, n=79 were primary spontaneous pneumothorax) and 31.8% (n = 41) of patients were traumatic pneumothorax (21.95% were iatrogenic pneumothorax). Main complaint is shortness of breath (52.3%, n=67) and 38% (n=49) of patients were smokers. Posteroanterior (PA) Chest X-Ray has been enough for 64.3% (n = 83) of the patients' diagnosis. Tube thoracostomy is applied to 84.5% (n = 109) of patients and surgery is applied to 9.3% (n = 12) of patients and 6.2% (n = 8) of patients were discharged with conservative treatment. Spontaneous pneumothorax showed statistically significant high recurrence compared with traumatic pneumothorax (P = 0.007). 4.65% of (n = 6) patients died. The average age of those who died (9.3 ± 19.9), statistically were significantly lower the mean age of living patients (32.4 ± 19.7) (t test, P = 0,006). 83.33% of the patients who died were neonatals and in the 0-1 years age group, and five of these patients were secondary spontaneous pneumothorax, and one of these patients were iatrogenic pneumothorax due to mechanical ventilation.

**Conclusions:**

Pneumothorax in adults can be treated by tube thoracostomy or surgically. Despite treatment, mortality of secondary and iatrogenic pneumothorax in newborns and 0-1 years age group is high.

## 1. Background

More than half of pneumothorax cases are spontaneous pneumothorax that occurs without any trauma (1-3). The pneumothorax developed without trauma or lung disease is called primary spontaneous pneumothorax (PSP), and occurred as a result of underlying lung disease such as chronic obstructive lung disease known as a secondary spontaneous pneumothorax (SSP) (4-6). Those consisting of reasons such as thoracentesis, biopsies and central venous catheterization is called iatrogenic pneumothorax, and consisting of post-traumatic reasons such as chest trauma is called traumatic pneumothorax (3).

Pneumothorax is common and life-threatening clinical condition which may require emergency treatment in Emergency Medicine Departments. The patient’s complaint is usually associated with the area covered by pneumothorax and the patient's physiological reserve. Minor changes in lung volume builds no symptoms and not be able to detect during the inspection. The diagnosis of pneumothorax is about lines were seen around the visceral pleura surrounding the collapsed lungs on PA chest X-ray. Emergency treatment of pneumothorax is bed rest, oxygen therapy, observation, simple aspiration, closed intercostal tube drainage and tube thoracostomy.

## 2. Objectives

This article is planned retrospectively and discusses variety of workup and emergency treatments are carried out, and investigation of the pneumothorax association with gender, smoking, season and age in Emergency Medicine Departments where patients with pneumothorax applied first.

## 3. Material and Methods

This retrospective case-control and multi-center study was conducted in the patients treated with the diagnosis of pneumothorax in Okmeydani, Bakirkoy Dr. Sadi Konuk and Kartal Dr. Lutfi Kirdar Educational and Research Hospitals between 01.01.2010-31.12.2010. Okmeydani and Bakirkoy Dr. Sadi Konuk Educational and Research Hospitals are located Europan side of Istabul and one of the most important hospitals. Kartal Dr. Lutfi Kirdar Educational and Research Hospital is located Anatolian side of Istanbul. Each of all three hospitals is based on the main traffic routes and accepts a large number of patients with trauma. The average annual number of patient in the each of three hospital is close to each other and between 200.000 and 250.000. Socio-cultural structure of people who living around of these hospitals are similar and middle income levels. Number of graudate from universities are lower.

Patients diagnosed with pneumothorax in automation system were screened. All patients with complete recorded data were included to this study. Patients without complete recorded data were excluded. According to the etiology of the pneumothorax, study groups were arranged like spontaneous pneumothorax (PSP and SSP), and traumatic pneumothorax (general trauma-induced pneumothorax and iatrogenic pneumothorax). The pneumothorax ratio of patients calculated according to the size of pneumothorax by PA chest X-ray or thoracic computed tomography (CT) measured according to the American College of Chest Physicians (ACCP) guidelines, were taken as mean, minimum and maximum values. Light index was also used to calculate the diameter of pneumothorax. All analyses were performed with Statistical Package for the Social Sciences (SPSS) for Windows version 15.0, and the significance level adopted as P < 0.05. Chi-square test was used to compare groups for categorical variables, and to compare continuous variables the Student t test was used.

## 4. Results

Table 1 shows that 68.2 % (n = 88) of patients were spontaneous pneumothorax (61.36% were PSP) and 31.8% (n = 41) of patients were traumatic pneumothorax (21.95% were iatrogenic pneumothorax). There was no case of tension pneumothorax in all patient groups. 82.2% (n = 106) of patients was male and 17.8% (n = 23) of patients were female and mean age were 31.3 ± 20.2 (Minimum: 1, Maximum: 87). No significant difference was found between the mean age of spontaneous pneumothorax group (30.7 ± 21.0) and traumatic pneumothorax group (32,6 ± 18,7) (P = 0.629). 4.7% (n = 6) of patients were neonatal patients. 83.3% (n = 107) of patients in neonatal group were found to be of secondary spontaneous pneumothorax, and one patient were iatrogenic pneumothorax (due to mechanical ventilation). 12.1% (n = 16) of patients with traumatic pneumothorax were bilateral pneumothorax (three neonates and 0-1 years age group), and 2.27% (n = 3) of patients with spontaneous pneumothorax were bilateral pneumothorax (P = 0.033). Mean age of bilateral pneumothorax patients were 11.1 ± 12.2, mean age of unilateral pneumothorax patients were 32.52 ± 0,0 and the difference was statistically significant (P = 0.006). 

**Table 1.** Gender Distribution According to Pneumothorax Groups

	Pneumothorax Groups, No. (%)	Male, No. (%)	Female, No. (%)	Total
**SP^a^**		74 (69.81)	14 (60.86)	88
PSP^a^	54 (41.9)			
SSP^a^	34 (26.4)			
**TP** ^**a**^		32(30.19)	9 (39.14)	41
GTP^a^	32 (24.8)			
IP^a^	9 (7.0)			

^a^ abbreviations: SP: spontaneous pneumothorax, PSP: primary spontaneous pneumothorax, SSP: Secondary spontaneous pneumothorax, TP: traumatic pneumothorax, GTP: General Traumatic pneumothorax, IP: iatrogenic pneumothorax.

Patients with spontaneous pneumothorax were most commonly admitted to Emergency Medicine Departments with the complaint of shortness of breath, and the most common complaint of the patients with a diagnosis of traumatic pneumothorax were stab wounds. The most common cause of traumatic pneumothorax in patients with iatrogenic reasons was application of mechanical ventilation (Table 2). PA chest-X-Ray, and chest CT or both were used for diagnosis of patients (Table 3). 

**Table 2.** Complaints and Causes of Pneumothorax Cases

Complaints	Spontaneous Pneumothorax, No. (%)	Traumatic Pneumothorax, No. (%)
**Dispnea**	46 (52.3)	-
**Chest Pain**	9 (10.2)	-
**Dispnea and Chest Pain**	29 (33.0)	-
**Other**	4 (45)	-
**Stab wounds**	-	14 (34)
**Fall**	-	7 (17)
**Traffic Accident**	-	11 (27)
**Iatrogenic**	-	9 (22)
Thoracentesis	-	5 (55)
Central venous catheter	-	2 (22)
Mechanical ventilation	-	1 (11)
Intubation	-	1 (11)

**Table 3.** Distribution of Tests Based on the Etiology of Pneumothorax

Pneumothorax Groups	Diagnostic Tests			
	PA Chest X-Ray, No. (%)	Thorax CT, No. (%)	PA Chest X-Ray and Thorax CT, No. (%)	Total
**PSP^a^**	33 (25.58)	4 (31)	17 (13.17)	54 (41.86)
**SSP** ^**a**^	29 (22.84)	1 (0.77)	4 (3.1)	34 (26.35)
**TP** ^**a**^	13 (10.07)	5 (3.87)	14 (10.85)	32 (24.8)
**IP** ^**a**^	8 (6.2)	1 (0.77)	0 (0)	9 (6.97)

^a^ abbreviations: PSP: primary spontaneous pneumothorax, SSP: Secondary spontaneous pneumothorax, TP: traumatic pneumothorax, IP: iatrogenic pneumothorax.

38% (n = 49) of patients were smoker and 93.9% (n = 46) of these patients were male. The rate of males who smoke was significantly higher than the rate of females who smoke (P = 0,007). 60.2% (n = 53) of spontaneous pneumothorax patients and 65.9% (n = 27) of traumatic pneumothorax patients were non-smoker.

There were no significant difference between smoking and pneumothorax type (P = 0.072) (Table 4). ACCP guidelines defines the size of pneumothorax as centimeters measured from the cupula to apex of lung according to PA Chest-X-ray or chest CT of patients, the mean of spontaneous pneumothorax were 3.8 ± 2.7 cm and traumatic pneumothorax were 2.2 ± 1.9 cm (P < 0.001). The mean of 32 patients with general trauma which related from traumatic pneumothorax cases were 2.4 ± 2.1 cm, and the mean of the group iatrogenic pneumothorax was found 1.63 ± 1.1 cm (P = 0.396). According to age group, the percentage of spontaneous pneumothorax group were 76%, and traumatic pneumothorax group was calculated as 52%, using Light index which defines the average of diameter of hemithorax as 10 cm. 

**Table 4.** Comparison of Men and Women, Smoking Status and Pneumothorax

	No Smoking, No. (%), n = 80	Smoking, No. (%), n = 49	Total
**Gender**			
Male	60 (75)	46 (93.9)	106
Female	20 (25)	3 (6.1)	23
**Pneumothorax Group**			
SP^a^	53 (60.2)	35 (39.8)	88
TP^a^	27 (65.9)	14 (34.1)	41

^a^ abbreviations: SP: spontaneous pneumothorax, TP: traumatic pneumothorax.

The tube thoracostomy is applied to the 84.5% (n = 109) of patients in the study group, and 6.2% (n = 8) were followed up tubeless. Surgical procedure performed to the 9.3% (n = 12) of the patients. The average duration of remaining the tube of the 109 patients treated by tube thoracostomy was found 5.6 ± 4.2 days. This duration were 5.8 ± 4.4 in spontaneous pneumothorax group, and 5.2 ± 3.8 days in traumatic pneumothorax group respectively (P = 0.384) (Table 5). Spontaneous pneumothorax recurrence developed in 20% (n = 18) of cases, and 2.4% (n=1) of the patients with traumatic pneumothorax had recurrence (P = 0.007). No cases of iatrogenic pneumothorax were developed recurrence. Recurrence rate of gender was 8.7% in female and 16% in male (P = 0.295). 

**Table 5.** Chest Tube Duration of Stay According to Etiology of Groups

Etiology	n	Chest Tube Duration of Stay	
		Mean (day)	Minimum – Maximum (day)
**PSP^a^**	54	4.9630	1.00 - 15.00
**SSP** ^**a**^	34	7.2353	1.00 - 22.00
**GTP** ^**a**^	32	4.8203	1.00 - 16.00
**IP** ^**a**^	9	6.3333	1.00 - 14.00
**Total**	129	5.6221	1.00 - 22.00

^a^ abbreviations: PSP: primary spontaneous pneumothorax, SSP: Secondary spontaneous pneumothorax, GTP: General Traumatic pneumothorax, IP: iatrogenic pneumothorax.

Six of 129 patients (4.65%) died during follow-up. Four of them were spontaneous pneumothorax group, and two of them were traumatic pneumothorax. Mortality in the patients with bilateral pneumothorax was observed in only one patient and mortality in the patients with unilateral pneumothorax were five (P = 0.289). The mean age of the patients who died were 9.3 ± 19.9, and the mean age of the living patients were 32.4 ± 19.7 (P = 0.006). Five of the six death cases (83.33%) were in neonatal group and 0-1 year’s age group.

## 5. Discussion

PSP shows variability in incidence because of different causes, and geography. In United States this incidence is 7.4-18/100000 in male, and 1.2-6/100000 in female (7, 8). The reason of PSP occurs in men more than women is the length of thorax is longer in men, apical blebs rupture due to increased pressure in apical region and excess rate of smoking as emphasized in the literatures (9). A 10-year follow up study by Gok M et al. (9) showed that, 12.2% of 164 spontaneous pneumothorax cases were female, and a study by Karasu S et al. (10) showed that 87.3% of 260 spontaneous pneumothorax case were men. Similar to the other studies the male predominance found in our study (82.2%).

The mean age of the patients were 27.03 ± 8.65 in the study by Karasu et al. (10). Similar to this study, the mean age of the patients was found 31.3 ± 20.2 in our study. Pneumothorax most commonly develops in the neonatal period after first breath in the first period of childhood (3, 11). Term infants and those patients with mechanical ventilation at high risk (12). SSP may develop those patients with asthma, pneumonia, lung disease such as cystic fibrosis (13). In this study 11.62% of patients were in age group one and 4.7% of patients were in neonatal group. Karasu et al. (10) were detected PSP in 91.5% of patients and significant difference was found between these patients and SSP according to age, gender, smoking, duration of treatment, and recurrence. However, in this study 41.9% of the patients were PSP.

In the vast majority of cases of pneumothorax most common symptom is sudden chest pain localized side of pneumothorax in emergency services. The second most common presenting symptom is shortness of breath. In Celik et al. (14) study, the most frequent symtom was shortness of breath (52.1%), and then after chest pain (20.8%), shortness of breath with chest pain (15.6%) and cough (9.3%) followed. Similar to the literature, in this study most frequent symptom is shortness of breath (52.3%), and shortness of breath with chest pain (33.0%). In the study of Imamoglu et al. (15), 110 patients with thoracic trauma were 59.1% blunt trauma, 36.4% penetrating trauma (25.45% stab and 10.90% gunshot wounds), and 4.5% presented with iatrogenic trauma. In this study, similar to Imamoglu et al. study, 26.8% of patients with traumatic pneumothorax are in stab wounds; but different from the literature, 21.95% of patients with traumatic pneumothorax were iatrogenic pneumothorax. Standard X-ray, ultrasound and chest CT can be used for diagnosis. For stable patients PA chest X-ray is standard although its sensitivity is 83% (16). Karasu et al. (10) were used X-Ray in 98% of patients and CT in 2% of patients to diagnose. In this study, PA chest X-Ray is only follow-up for 64.3% of cases with pneumothorax. Ultrasonography was not used in Emergency Department for diagnosis of pneumothorax.

Pneumothorax rate of all life-long smokers is approximately 12%, meanwhile 0.1% for non-smokers (17). In Karasu et al. (10) study smoking rate was 86.9%. In the patients with PSP and SSP rate of smoking was 93.3% and 18.2%, respectively. In this study 39.8% of spontaneous pneumothorax was smokers. 93.9% of smoker patients in the study were male, and it is an indication that men are more smoker than women in our country. In the study of Topdag et al. mean percentage of pneumothorax were 71%. In this study the average size of spontaneous pneumothorax, was significantly higher than the average size of traumatic pneumothorax. According to Light index group percentages of pneumothorax at spontaneous pneumothorax (76%) were higher than traumatic pneumothorax (52%).

In the study on 592 trauma patients of Tekinbas et al. 158 patients were pneumothorax, and 119 patients were hemothorax. 57.26% of the patients were aplied tube thoracostomy, 99 patients were applied surgical treatment, and 190 patients were treated conservatively, and the mean hospital stay was 13.4 days with 6.4% mortality rate. According to study of Wolfman et al. many small occult pneumothoraces can be observed by close follow-up, most of them have low progression risk, and intermediate-size anterior occult pneumothoraces initially managed with observation if positive-pressure ventilation was not anticipated also did not require chest tube placement. Tube thoracostomy is recommended to anterolateral occult pneumothorax cases. In this study, tube thoracostomy is applied to 79.6% of patients and surgery is applied to 9.3% of patients and 6.2% of patients were discharged with conservative treatment. On all patients performed tube thoracostomy in the emergency department.

In the 266 case study of Burcin et al. (14) the mean duration of hospitalization was 9.3 ± 5.3 days and there was significant difference between groups of PSP and SSP and duration of hospitalization. In the 53 case study of Cok et al. (18) the mean duration of hospitalization was 7.7 ± 3.2 days in PSP group and 23.2 ± 18.6 days were in SSP group. In the same study (18), closed intercostal tube drainage applied to 17 patients in primary spontaneous pneumothorax group (mean 7.1 ± 3.3 days) and to 22 patients in SSP group (mean 20 ± 14.3 days). In Topdag et al. study, tube thoracostomy performed to all patients for treatment and the average length of stay of tube determined as 4.6 days. In this study, the average length of stay of tube of 109 patients that tube thoracostomy were performed was 5.6 ± 4.2 days, less than literature.

Recurrence rate of PSP is 32% and of SSP is 43%, if there is not any prevention for recurrence 6.7. Iatrogenic pneumothorax recurrences in the long term are uncommon (19). In Karasu et al. (10) study 8.5% recurrence was observed (7.6% for PSP and 22.7% for SSP). In Kuzucu et al. (20) comparative study with 90 PSP cases between 1999-2004 reported that 17 patients were applied surgery, 24 patients of remaining 73 patients developed recurrence, 15 of the patients with recurrence were applied surgery in second or third episodes, and surgical treatment is best choice for second episode. In this study recurrence was observed in 20% cases of spontaneous pneumothorax and 2.4% cases of traumatic pneumothorax. No recurrence for iatrogenic pneumothorax was found with respect to the literature. 1.3% of cases of spontaneous pneumothorax has been reported that bilateral and simultaneous spontaneous pneumothorax (21). Eleven countries identified 1988 cases of pneumothorax, and they showed that 1.3% of cases were simultaneous bilateral spontaneous pneumothorax (22). Between 1971-1990 Esther et al. reported 12 case of simultaneous bilateral spontaneous pneumothorax. In this study, bilateral pneumothorax detected in 5.4% of pneumothorax cases and 2.27% of spontaneous pneumothorax cases.

Mortality of PSP is less than 1% because most of patients are young (23, 24). Mortality of PSP is getting higher due to advanced lung disease and increased physiological reserves (23, 24). A study with 83 cases by Ilce et al. showed that the relationship between mortality and an underlying primary lung disease, low birth weight, prematurity and mechanical ventilation therapy were statistically significant. In the study with 53 cases by Cok et al. (18) 7.14% of deaths from 28 patients with SSP have been reported. In this study, spontaneous pneumothorax mortality were 3.11%, and 1.55% were in traumatic pneumothorax. In this study, the average age of the patients resulting in mortality, was lower than not resulting in mortality significantly. 83.33% of the patients who died were neonatal and in the age group one, and five of these patients were secondary spontaneous pneumothorax, and one of these patients were iatrogenic pneumothorax due to mechanical ventilation. Pneumothorax in adults can be treated by tube thoracostomy or surgically. Despite treatment, mortality of secondary and iatrogenic pneumothorax in newborns and 0-1 year’s age group is high. It should be more carefully in management of less than 1 year’s age group.

## References

[1] Light RW, Light RW (2007). Pneumothorax.. Pleural Diseases..

[2] Noppen Marc, De Keukeleire Tom (2008). Pneumothorax.. Respiration..

[3] Noppen M, De Keukeleire T (2008). Pneumothorax.. Respiration..

[4] Buscom R (2011.). Pneumothorax..

[5] Yegen BÇ, Çavuşoğlu H, Çakır L (2006). Guyton&Hall.

[6] Sadikot RT, Greene T, Meadows K, Arnold AG (1997). Recurrence of primary spontaneous pneumothorax.. Thorax..

[7] Guo Y, Xie C, Rodriguez RM, Light RW (2005). Factors related to recurrence of spontaneous pneumothorax.. Respirology..

[8] Edenborough FP, Hussain I, Stableforth DE (1994). Use of a Heimlich flutter valve for pneumothorax in cystic fibrosis.. Thorax..

[9] Gök Mehmet, Ceran Sami, Sunam Güven, Uzun Kürşat (2007). [Spontan pnömotorakslı kadın olguların değerlendirilmesi].. Tıp Araştırmaları Dergisi..

[10] Karasu Sezgin, Tokat ArifOsman, Kısacık Erkan, Çakmak Hüseyin, Karakaya Jale, Aydın Ertan (2012). Spontaneous pneumothorax: analysis of 260 patients.. J Clin Anal Med..

[11] Al Tawil Khalil, Abu‐Ekteish FaisalM, Tamimi Omar, Al Hathal MuneefM, Al Hathlol Khalid, Abu Laimun Bdeir (2004). Symptomatic spontaneous pneumothorax in term newborn infants.. Pediatric Pulmonology..

[12] Sahn StevenA, Heffner JohnE (2000). Spontaneous pneumothorax.. N Engl J Med..

[13] Flume PatrickA (2009). Pulmonary complications of cystic fibrosis.. Respir Care..

[14] Çelik Burçin, Furtun Kamil, Demir Hasan, Yılmaz MAli (2009). [Spontan pnömotorakslı olgularımızın klinik özellikleri].. Gülhane Tıp Dergisi..

[15] Imamoğlu Oya. Uncu, Öncel Mustafa, Erginel Turgay, Tunçay Erhan, Dalkiliç Gülay, Acar Hakan (1999). Toraks Travmalarinda Yaklaşim: 110 Olgunun Değerlendirilmesi.. Türk Göğüs Kalp Damar Cerrahisi Dergisi..

[16] Seow Albert, Kazerooni EllaA, Pernicano PG, Neary Maureen (1996). Comparison of upright inspiratory and expiratory chest radiographs for detecting pneumothoraces.. AJR Am J Roentgenol..

[17] Bense Laszlo, Eklund Gunnar, Wiman Lars-Gosta (1987). Smoking and the increased risk of contracting spontaneous pneumothorax.. Chest..

[18] ÇOK Gürsel, KARAKUŞ Haydar, GÖKSEL Tuncay, GÜZELANT Asuman, BAYINDIR Ülkü (2001). Primer ve sekonder spontan pnömotorakslı olguları karşılaştıran geriye dönük bir çalışma.. Toraks Dergisi..

[19] Laronga Christine, Meric Funda, Truong MyleneT, Mayfield Carla, Mansfield Paul (2000). A treatment algorithm for pneumothoraces complicating central venous catheter insertion.. Am J Surg..

[20] Henry M, Arnold T, Harvey J (2003). BTS guidelines for the management of spontaneous pneumothorax.. Thorax..

[21] Melton 3rd LJ, Hepper NG, Offord KP (1979). Incidence of spontaneous pneumothorax in Olmsted County, Minnesota: 1950 to 1974.. Am Rev Respir Dis..

[22] Graf-Deuel Esther, Knoblauch Andreas (1994). Simultaneous bilateral spontaneous pneumothorax.. Chest..

[23] Melton LJ, Hepper NG, Offord KP (1979). Incidence of spontaneous pneumothorax in Olmsted County, Minnesota: 1950 to 1974.. Am Rev Respir Dis..

[24] Gupta Dheeraj, Hansell Anna, Nichols Tom, Duong Trinh, Ayres JonG, Strachan David (2000). Epidemiology of pneumothorax in England.. Thorax..

